# Whole-genome sequence analysis reveals selection signatures for important economic traits in Xiang pigs

**DOI:** 10.1038/s41598-022-14686-w

**Published:** 2022-07-12

**Authors:** Xiying Wang, Xueqin Ran, Xi Niu, Shihui Huang, Sheng Li, Jiafu Wang

**Affiliations:** 1 Institute of Agro-Bioengineering/Key Laboratory of Plant Resource Conservative and Germplasm Innovation in Mountainous Region and Key Laboratory of Animal Genetics, Breeding and Reproduction in the Plateau Mountainous Region (Ministry of Education), College of Life Science and College of Animal Science, Guizhou University, Guiyang, 550025 China; 2 Tongren University, Tongren, 554300 China

**Keywords:** Computational biology and bioinformatics, Evolution, Genetics, Molecular biology

## Abstract

Xiang pig (XP) is one of the best-known indigenous pig breeds in China, which is characterized by its small body size, strong disease resistance, high adaptability, favorite meat quality, small litter sizes, and early sexual maturity. However, the genomic evidence that links these unique traits of XP is still poorly understood. To identify the genomic signatures of selection in XP, we performed whole-genome resequencing on 25 unrelated individual XPs. We obtained 876.70 Gb of raw data from the genomic libraries. The LD analysis showed that the lowest level of linkage disequilibrium was observed in Xiang pig. Comparative genomic analysis between XPs and other breeds including Tibetan, Meishan, Duroc and Landrace revealed 3062, 1228, 907 and 1519 selected regions, respectively. The genes identified in selected regions of XPs were associated with growth and development processes (*IGF1R*, *PROP1*, *TBX19*, *STAC3*, *RLF*, *SELENOM*, *MSTN*), immunity and disease resistance (*ZCCHC2*, *SERPINB2*, *ADGRE5*, *CYP7B1*, *STAT6*, *IL2*, *CD80*, *RHBDD3*, *PIK3IP1*), environmental adaptation (*NR2E1*, *SERPINB8*, *SERPINB10*, *SLC26A7*, *MYO1A*, *SDR9C7*, *UVSSA*, *EXPH5*, *VEGFC*, *PDE1A*), reproduction (*CCNB2*, *TRPM6*, *EYA3*, *CYP7B1*, *LIMK2*, *RSPO1*, *ADAM32*, *SPAG16*), meat quality traits (*DECR1, EWSR1*), and early sexual maturity (*TAC3*). Through the absolute allele frequency difference (ΔAF) analysis, we explored two population-specific missense mutations occurred in *NR6A1* and *LTBP2* genes, which well explained that the vertebrae numbers of Xiang pigs were less than that of the European pig breeds. Our results indicated that Xiang pigs were less affected by artificial selection than the European and Meishan pig breeds. The selected candidate genes were mainly involved in growth and development, disease resistance, reproduction, meat quality, and early sexual maturity. This study provided a list of functional candidate genes, as well as a number of genetic variants, which would provide insight into the molecular basis for the unique traits of Xiang pig.

## Introduction

Pigs (*Sus scrofa*) are important agriculture animals with an ancient domestication history and economic values and have become an important source of protein for human food. Moreover, the pig serves as a valuable biomedical model for some specific human diseases such as obesity and cardiovascular diseases^1,2^. Pigs from the original European and Asian wild boars were independently domesticated approximately 10,000 years ago^3^. From early domestication to modern breeding practices, pigs have undergone a series of natural and artificial selections in various environments which have resulted in high levels of phenotypic diversity in morphological, physiological and behavior traits. There are more than 730 pig breeds or lines worldwide, two thirds of which are distributed in China and Europe. These pig breeds show different characteristics with respect to size, color, body shape, ear carriage, behavior, prolificacy, as well as other traits. For example, European domestic Duroc and Yorkshire pigs are best known for their large body size, fast growth rate, larger feed conversion ratio, superior meat yield, and slaughter rate^4^. Both of them have been experienced very strong selection. Meishan pigs in East China are characterized by their high fecundity, wrinkled black skin, large drooping ears, and early maturity^5^. Tibetan pigs, a representative of Asian wild boar, have characteristics of small body size, low litter size, strong adaptability and body-resistance^6^. Whole genome-wide scans of diverse pig breeds can increase our understanding of the manner in which genomic variation has been shaped and how the advantageous characteristics have been evolved through artificial selection. With the development and application of next-generation sequencing techniques, increasing studies have been conducted to unveil the genetic mechanisms underlying the phenotypes of pigs^5,6^.

As a major center of early pig domestication, China possesses 88 indigenous pig breeds with unique characteristics^7^. Because of the diverse phenotypes among those pig breeds, the genetic for the phenotypic variations remain unknown, particularly for the indigenous Xiang pig breeds. Xiang pig (XP) is a representative indigenous mini-pig breed in China. Besides their small body size, this pig breed has characteristics of strong disease resistance, high adaptability, favorable meat quality, low litter sizes, and early sexual maturity^8,9^. The unique features of Xiang pig were evolved through long-time natural and artificial selections. Our previous studies have reported the copy number variations (CNVs) and structural variations (SVs) of Xiang pig by Porcine SNP60K BeadChip and resequencing approaches, respectively and identified a lot of genes invovled in growth and repoduction^8,9^. Based on RNA sequencing, we have also identified some genes which played an important role in estrous and litter size in female Xiang pigs^10,11^. However, the molecular mechanisms underlying the breed characteristics in Xiang pigs remain unclear.

In this study, we performed whole-genome resequencing on 25 unrelated individual XPs. Given the phenotypic and genomic differences between Xiang pig and Tibetan, Meishan, Duroc, and Yorkshire, we then separately conducted the first comparative genomic analysis between XPs and each of the pig breeds and sought to identify the genomic signatures of selection in XPs. We explored the genomic regions under selection in XPs using three complementary methods: Fst, θπ and the absolute allele frequency difference (ΔAF). We identified many heritable variants and a suite of potential candidate genes that are involved in crucial biological processes such as growth, disease resistance, reproduction, meat quality traits and early sexual maturity. These findings will provide useful information to improve our understanding of the molecular mechanisms underlying the unique traits of XPs.

## Results

### Characteristics of the genome datasets

Whole-genome resequencing yielded a total of 876.70 Gb raw paired-end reads from the 25 unrelated individual XPs. Within this data, 819.09 Gb (93.43%) of high-quality paired-end reads were obtained after strict quality control protocols. Subsequently, the high-quality clean reads were then aligned to the pig reference genome assembly (Sscrofa11.1), resulting in a mean mapping rate of 96.91%. The average sequencing depth for each sample was 12.98 × (from 11.60 × to 19.88 ×) (TableS1). To accurately detect genomic footprints, we obtained the publicly available whole genome data of 100 individuals pigs from NCBI database, including Tibetan pig (TT, n = 25), Meishan (MS, n = 25), Duroc (DU, n = 25) and Yorkshire (LW, n = 25). These downloaded datasets had a total of 3661 Gb raw reads. The average depth of the downloaded datasets was 12.93 × in the 100 publicly available pigs (TableS2).

### Identification of heritable variants

**Figure 1:**
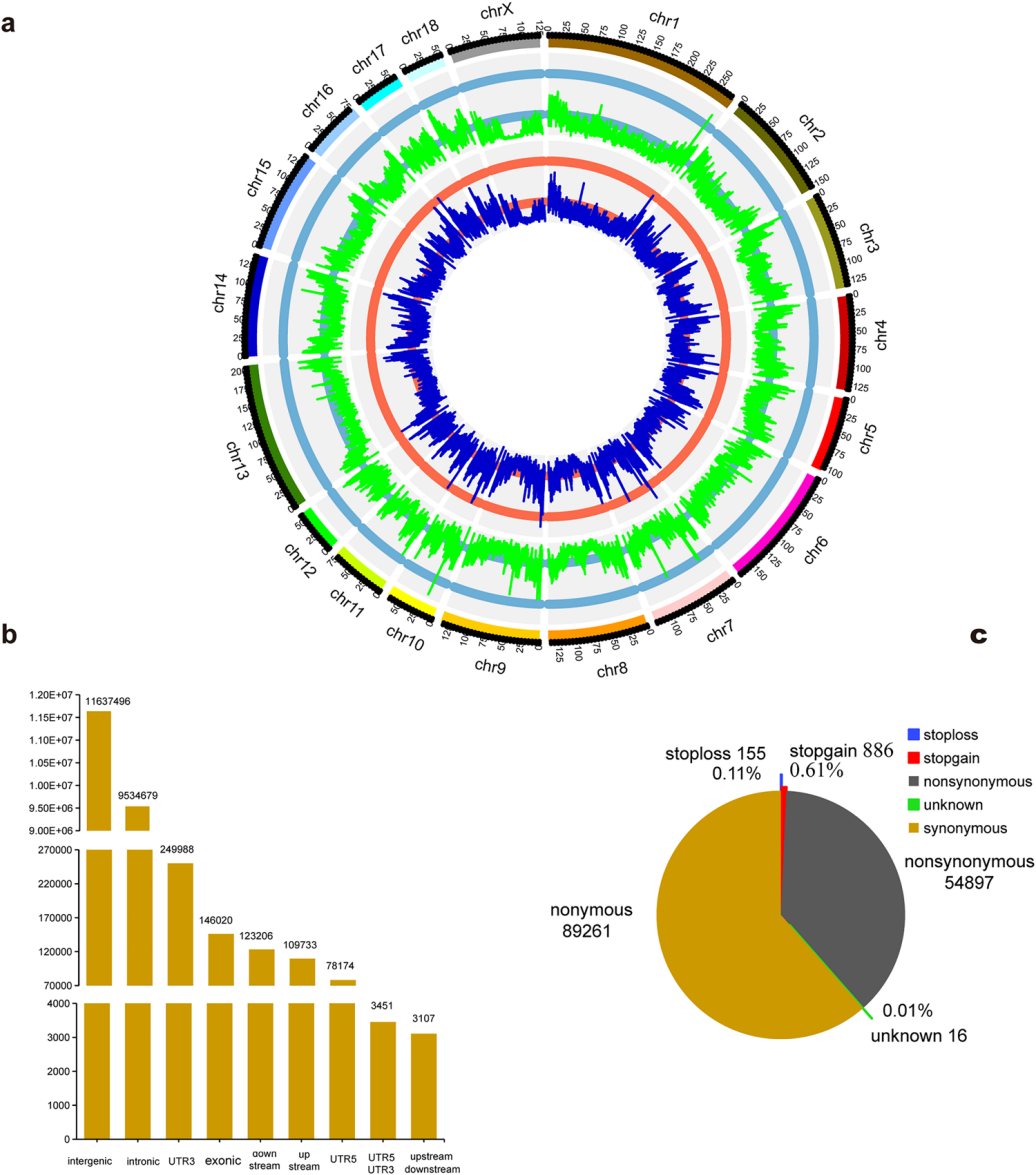
The distribution and annotation of genomic variants in XP. (**a**) Circos plot of distribution of variants density after filtration. The density of variants was calculated in each 100 kb step size. The circles from inside to outside display INDEL and SNP density per window, respectively. (**b**) SNP numbers in genomic annotation according to ANNOVAR. (**c**) The pie plot shows annotated SNPs at exonic regions.

In total, 21,885,854 SNPs and 5,761,765 INDELs were obtained from Xiang pig genome (Table 1). Figure 1a showed the densities of SNPs and INDELs on each chromosome of XPs. We found that the maximum density of SNPs and INDELs was located on chromosomes 10 and 7, respectively. Of the total SNPs in XPs, 18,375,288 SNPs (83.96%) were identified in the dbSNP database, and 3,510,566 (16.04%) SNPs were newly found. Compared with other four pig breeds, 3,985,444 variants in our data set are Xiang-specific SNPs (Fig. S1).

**Table 1:** Summary of the functional annotation statistics of SNPs in XP, TT, MS, DU and LW.

SNP categories	XP	TT	MS	DU	LW
Upstream	109,733	101,205	63,085	53,181	45,588
UTR5	78,174	70,823	44,755	37,616	32,244
**Exonic**					
Nonsynonymous	54,897	47,693	32,950	28,676	22,030
Synonymous	89,261	78,034	53,931	43,824	37,320
Stopgain	886	769	520	397	298
Stoploss	155	151	85	98	70
Unknown	16	20	13	14	16
Splicing	805	725	469	421	379
Intronic	9,534,679	9,463,488	5,866,342	4,765,630	4,637,511
Downstream	123,206	242,955	152,657	125,375	117,879
Upstream/downstream	3107	3077	2017	1472	1324
UTR3	249,988	119,609	74,922	62,124	56,394
UTR5;UTR3	3451	2772	1536	1336	1224
Intergenic	11,637,496	11,770,026	7,300,453	6,046,060	5,739,841
Numbers of total SNP	21,885,854	21,901,347	13,593,735	11,166,224	10,692,118

XP, Xiang pig; TT, Tibetan; MS, Meishan; DU, Duroc; LW, Yorkshire.

Further functional annotation of these identified SNPs in the Xiang genome revealed that SNPs were partitioned into intergenic (11,637,496; 53.17%), intronic (9,534,679; 43.57%), exonic (145,215; 0.66%) and other gene regulatory regions (Table 1, Fig. 1b). In exonic regions, most variants resulted in synonymous (89,261) and nonsynonymous (54,897) mutations, followed by stop gain, stop loss, and unknown (< 1%) (Fig. 1c).

### Population genetic structure and linkage disequilibrium

**Figure 2:**
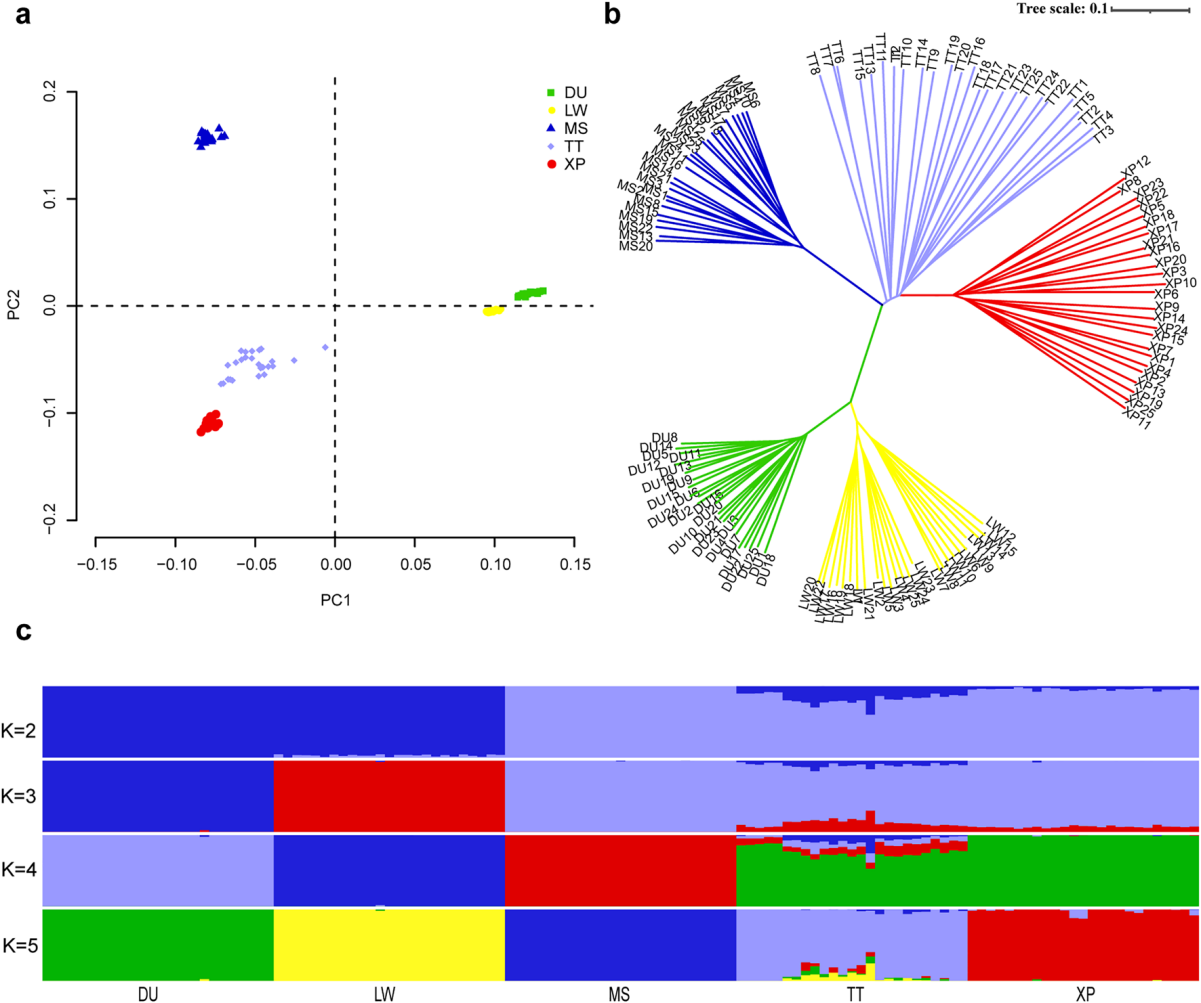
Population genetic analysis. (**a**) Plots of principal components 1 and 2 for the 125 individuals. (**b**) Neighbor-joining tree constructed from SNPs data among five pig populations. (**c**) Genetic structure analysis of samples using Admixture, with changing ancestral populations from K = 2 to K = 5.

**Figure 3:**
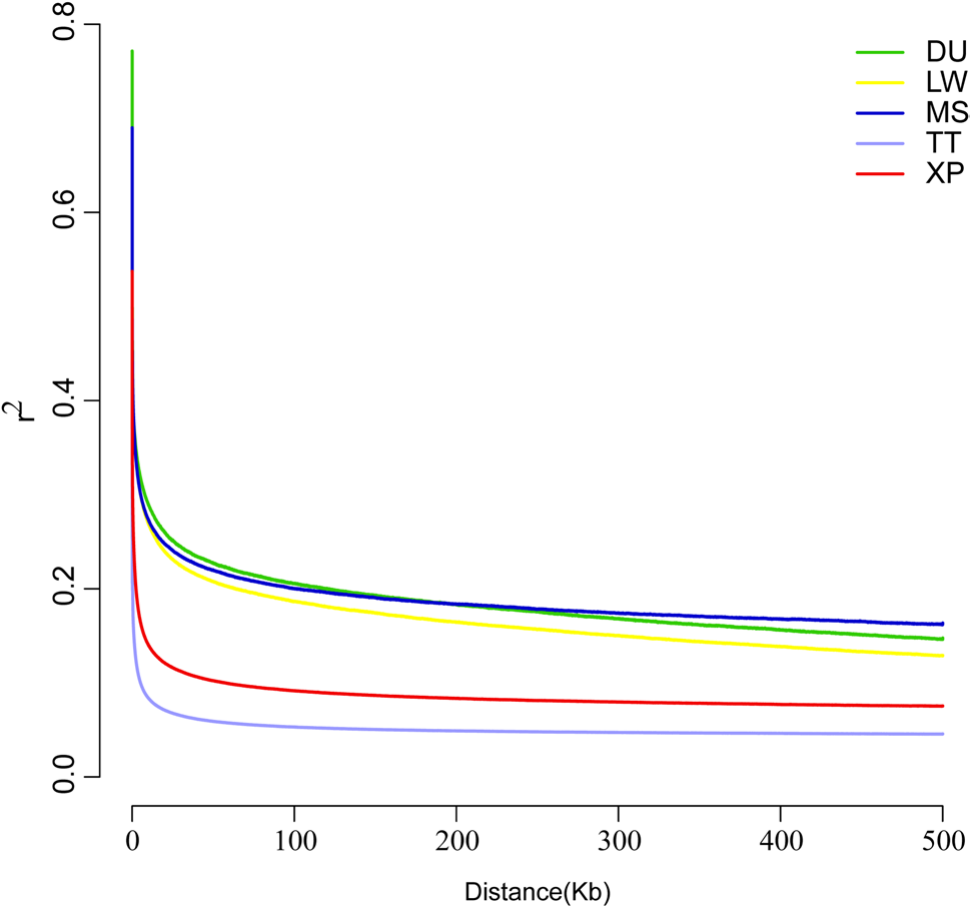
Decay of linkage disequilibrium in XP, TT, MS, DU and LW breeds.

To assess the genetic structure among the pig breeds in this study, principal component analysis (PCA), neighbor-joining (NJ) trees, and ADMIXTURE were performed. The analysis of PCA (Fig. 2a) revealed that the whole pig population was divided into five groups: XP, TT, MS, DU and LW. The first component (PC1) separated the European-originated pig (DU and LW) from Chinese pig (XP, TT and MS). The second component separated XP and TT from MS. Meanwhile, DU and LW were divided by PC2. The branches of the phylogenetic tree (Fig. 2b) were consistent with the results of PCA, and successfully divided into five populations displaying genetically distinct clusters. Further population admixture analysis (Fig. 2c) clearly distinguished two breeds as expected based on their origin at K = 2. At K = 3, the European pig populations were separated, whereas the Chinese populations were still clustered together. At K = 4, MS were separated from Chinese pig populations. A separate ancestry for all four populations was visible at K = 5. With respect to the LD comparison among five populations (Fig. 3), we observed similar LD decay rates in Meishan and Duroc populations and the lowest level of LD in TT breed. XP had a lower level of LD compared to MS, DU and LW (Fig. 3).

### Identification of selection signatures in XPs

**Figure 4:**
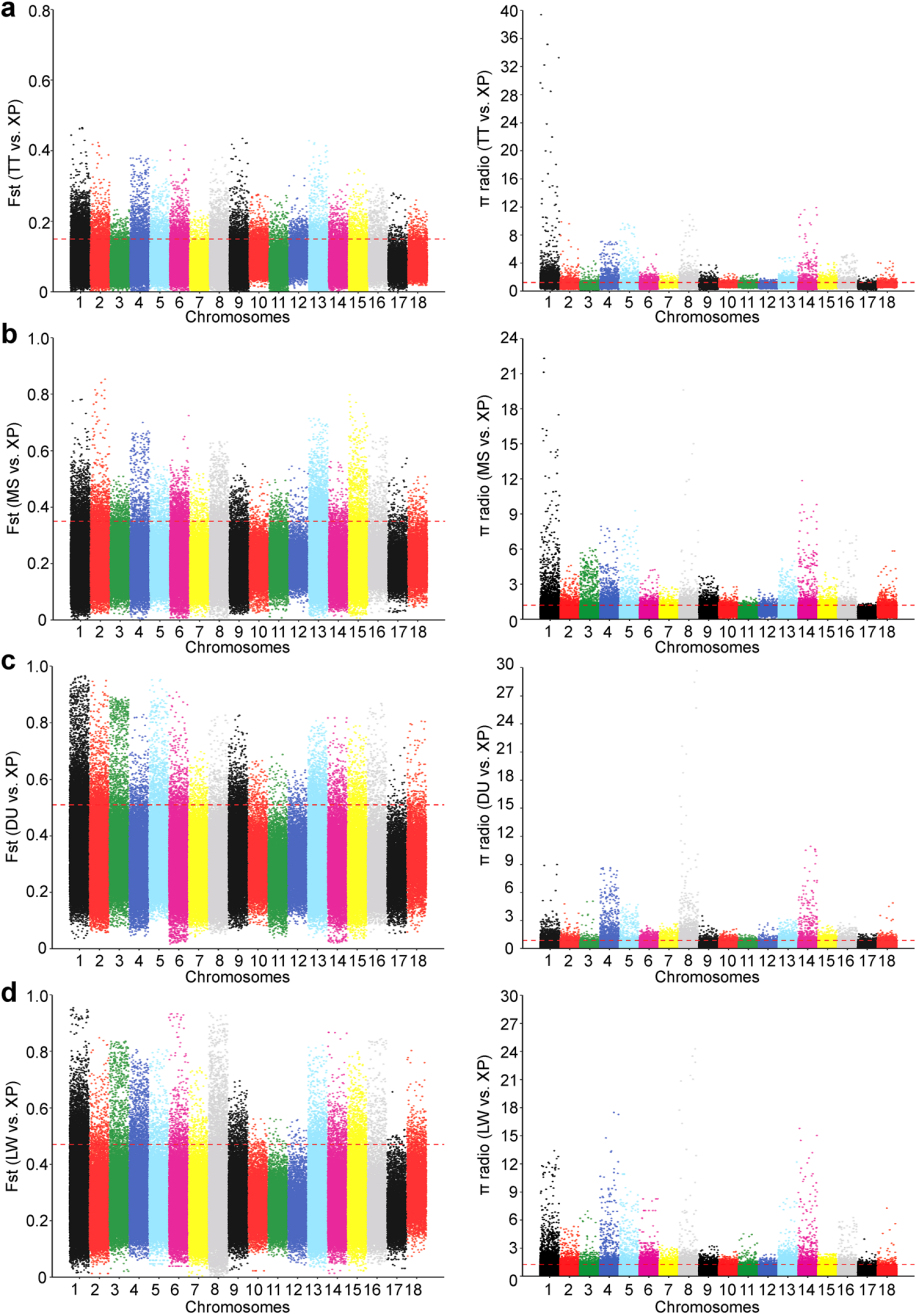
Manhattan plots of genome-wide of Fst and θπ values across all 18 autosomes identified in XP population from four different comparisons. (**a**) TT vs XP. (**b**) MS vs XP. (**c**) DU vs XP. (**d**) LW vs XP. Dashed lines represented the threshod of top 5% Fst and θπ values.

**Figure 5:**
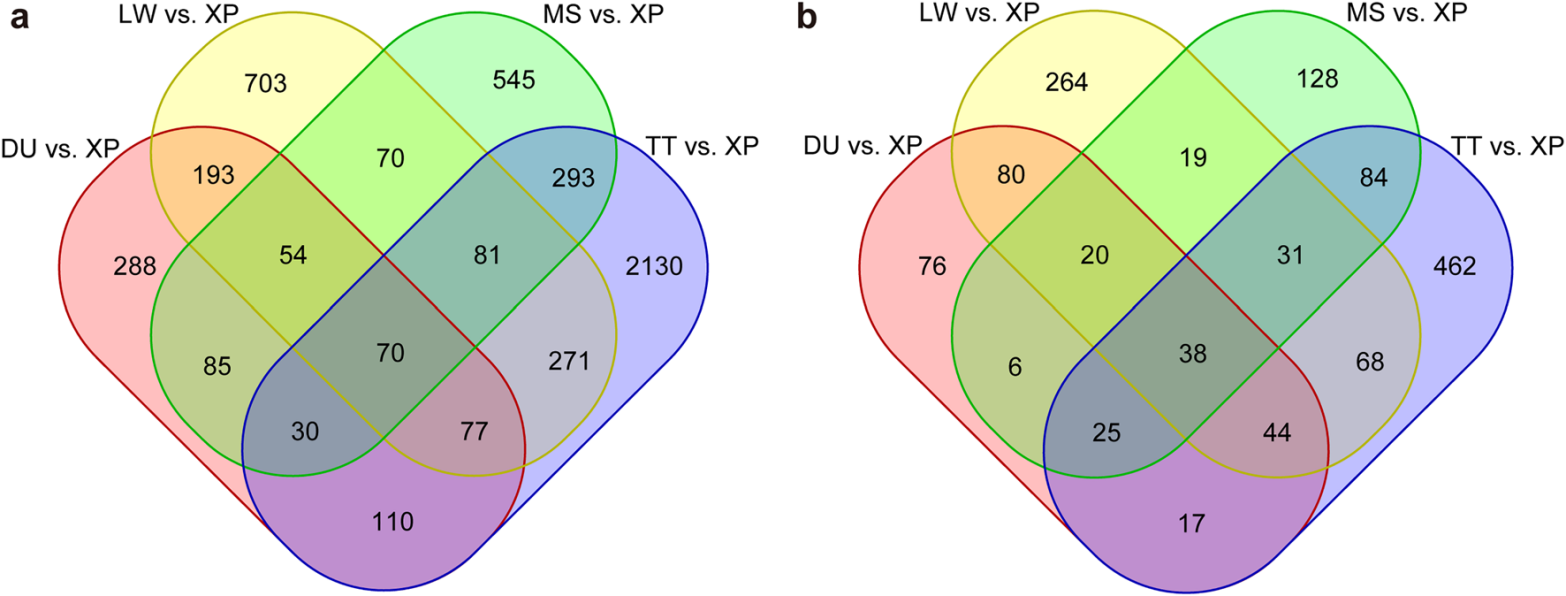
Venn diagrams represented the numbers of unique and shared (**a**) positively selected regions and (**b**) the candidate genes between four comparisons.

In order to detect the genome-wide selection signatures, we calculated pairwise Fst and θπ in 100-kb sliding windows with a step size of 10 kb across the autosomes between the XP and TT, MS, DU, and LW populations, respectively. For each comparison, the shared windows with both the Fst and θπ values in the top 5% were considered to be potentially positive selected regions. Based on the methods described above genome-wide screening revealed 3062 outlier windows as regions of selective sweeps with Fst > 0.15 and θπ > 1.23 in TT vs XP (Fig. 4a), 1228 outlier regions with Fst > 0.35 and θπ > 1.21 in MS vs XP (Fig. 4b), 907 outlier regions with Fst > 0.51 and θπ > 0.86 in DU vs XP (Fig. 4c), and 1519 outlier regions with Fst > 0.47 and θπ > 1.26 in LW vs XP (Fig. 4d). The numbers of the annotated genes within the selected regions were 769, 351, 306, and 564 in four comparative analyses (TT vs XP, MS vs XP, DU vs XP, and LW vs XP), respectively (Fig. 5b, Table S3). The numbers of unique and shared potential regions and candidate genes between four comparisons were shown in Fig. 5. We found that 38 candidate genes under selection were shared among all four comparisons (Fig. 5b, Table S4-1).

**Figure 6:**
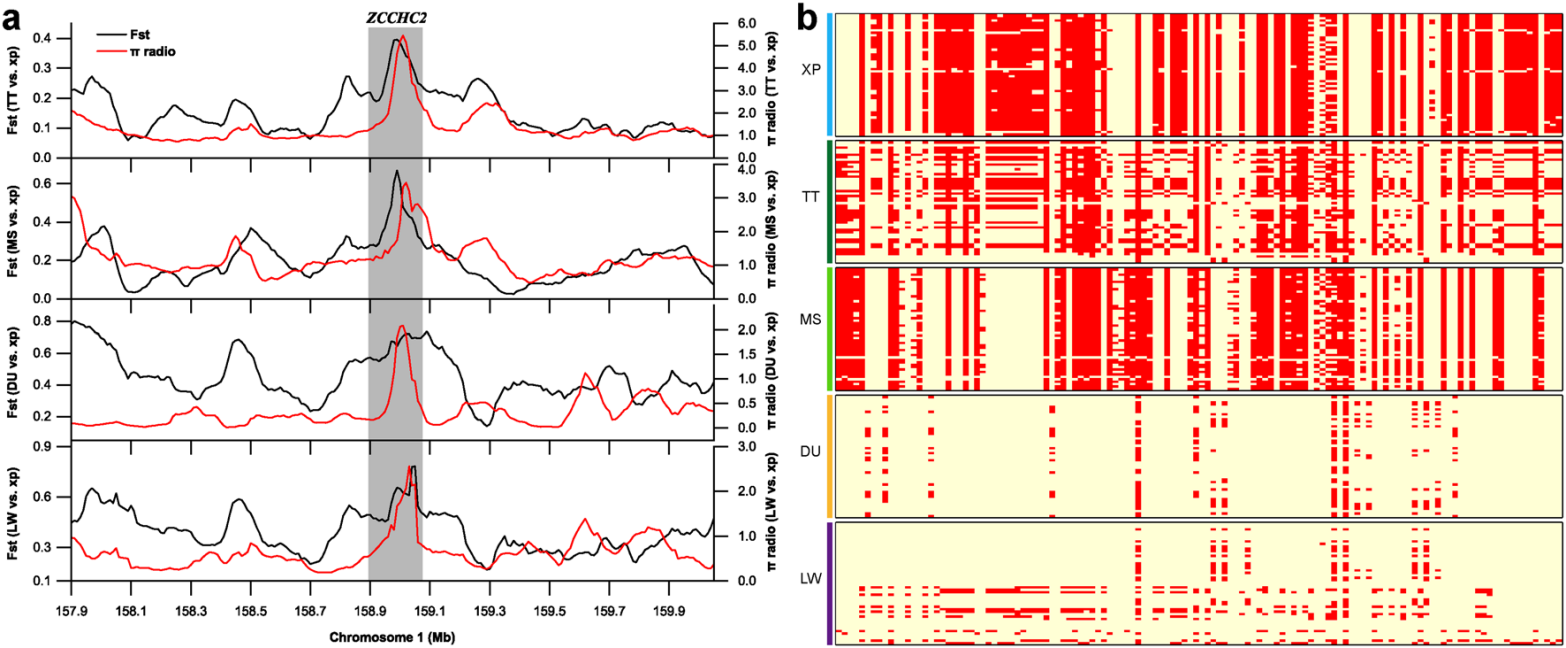
Characterization of selection signals around *ZCCHC2* gene locus in XPs. (**a**) The Fst and θπ values around the *ZCCHC2* locus. (**b**) Haplotype plots spanning *ZCCHC2* gene among the five pig populations. The allele consistent with reference genome was indicated in lightyellow and derived allele in red color.

**Figure 7:**
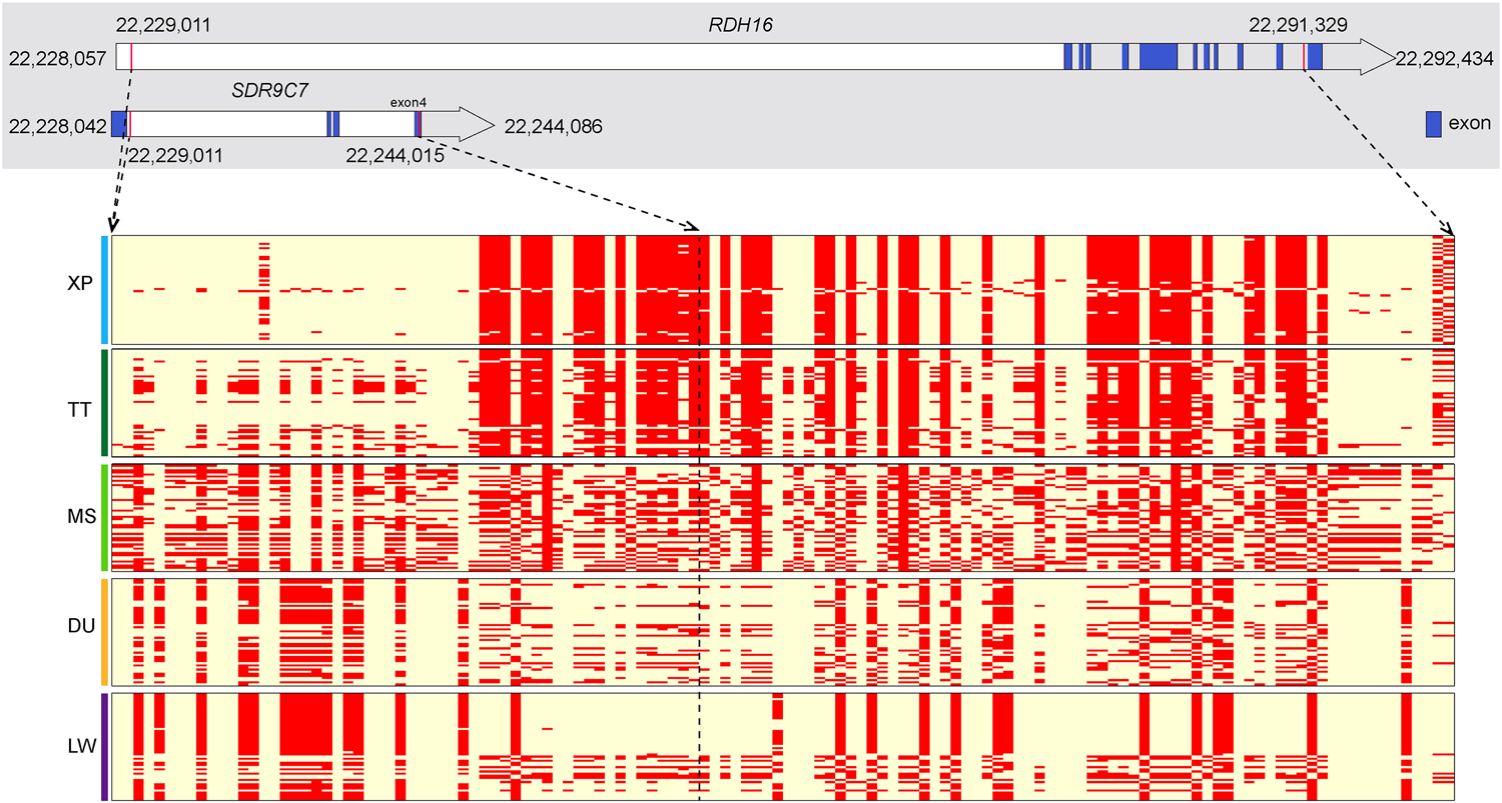
Haplotype plots spanning *SDR9C7* and *RDH16* genes among the five pig populations. The allele consistent with reference genome was indicated in lightyellow and derived allele in red.

**Figure 8:**
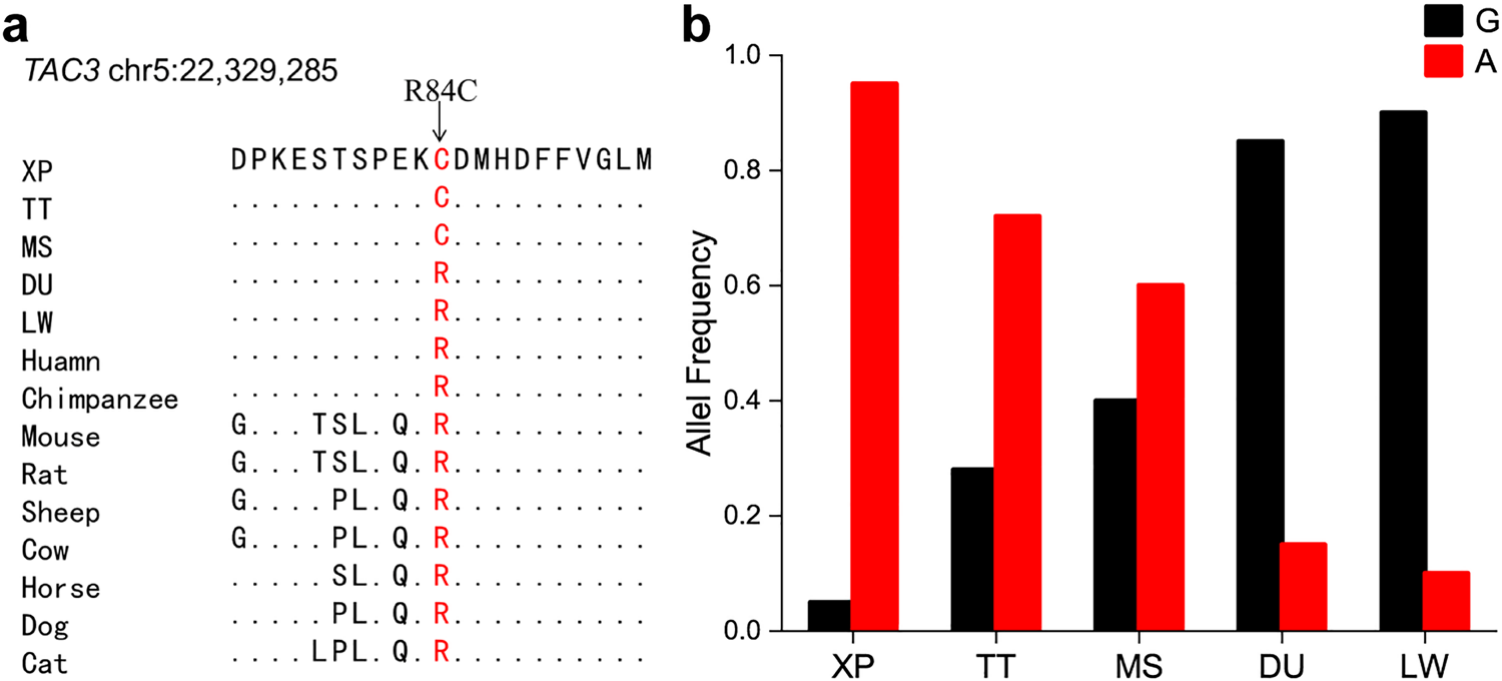
Putative functional variant (p.R84C) in *TAC3* gene. (**a**) Multispecies alignment of the protein sequences around the variant. The dots in the alignment indicated the amino acids which were identical with those in XP. (**b**) Distribution of the allele frequency of the variant in five pig populations.

Among the 38 co-selection genes, we observed that *ZCCHC2* on SSC1 showed strong selection signals. Both Fst and θπ ratios showed high values around the *ZCCHC2* locus in all four comparisons (Fig. 6a). Moreover, XP had different haplotype patterns with other four pig breeds (Fig. 6b), suggesting a strong selection for XP in this region. In addition, one gene on SSC4, five genes on SSC5 and one gene on SSC15 were selected for further research based on their known biological function, including *SLC26A7*, *SDR9C7*, *RDH16*, *TAC3*, *MYO1A*, *LYZ*, *PDE1A*. We found that the haplotype patterns of the *SDR9C7* and *RDH1*6 genes in XPs were different from that of the other four populations (Fig. 7), which suggested a strong selection for XP in these two genes. To analyze the signs of selection in detail, we detected the SNPs with highly significant effects, which located in exons, UTRs and downstream/upstream of the seven genes mentioned above. We found 36 SNPs with different mutation frequencies between the XP and the other four pig breeds in *SDR9C7*, *RDH16*, *TAC3*, *MYO1A*, *LYZ*, and *PDE1A* (Table S5). The genotype pattern of these SNPs showed significant differences among the five pig breeds (Fig. S2). We also noted that twelve of these 36 variants were nonsynonymous mutations (Table S6). Of note, a novel missense variant/splice variant (c.G895A; chr5:22,354,739) in frequency of the derived allele “A” was 0.98 in XPs (Table S6). In *TAC3*, one nonsynonymous variants (G > A; p.R84C; chr5:22,329,285) was predicted as functional-altering variant (SIFT = 0) (Table S6). The p.R84C variant in XPs was significantly different from MS, DU and LW populations (Fig. 8).

**Figure 9:**
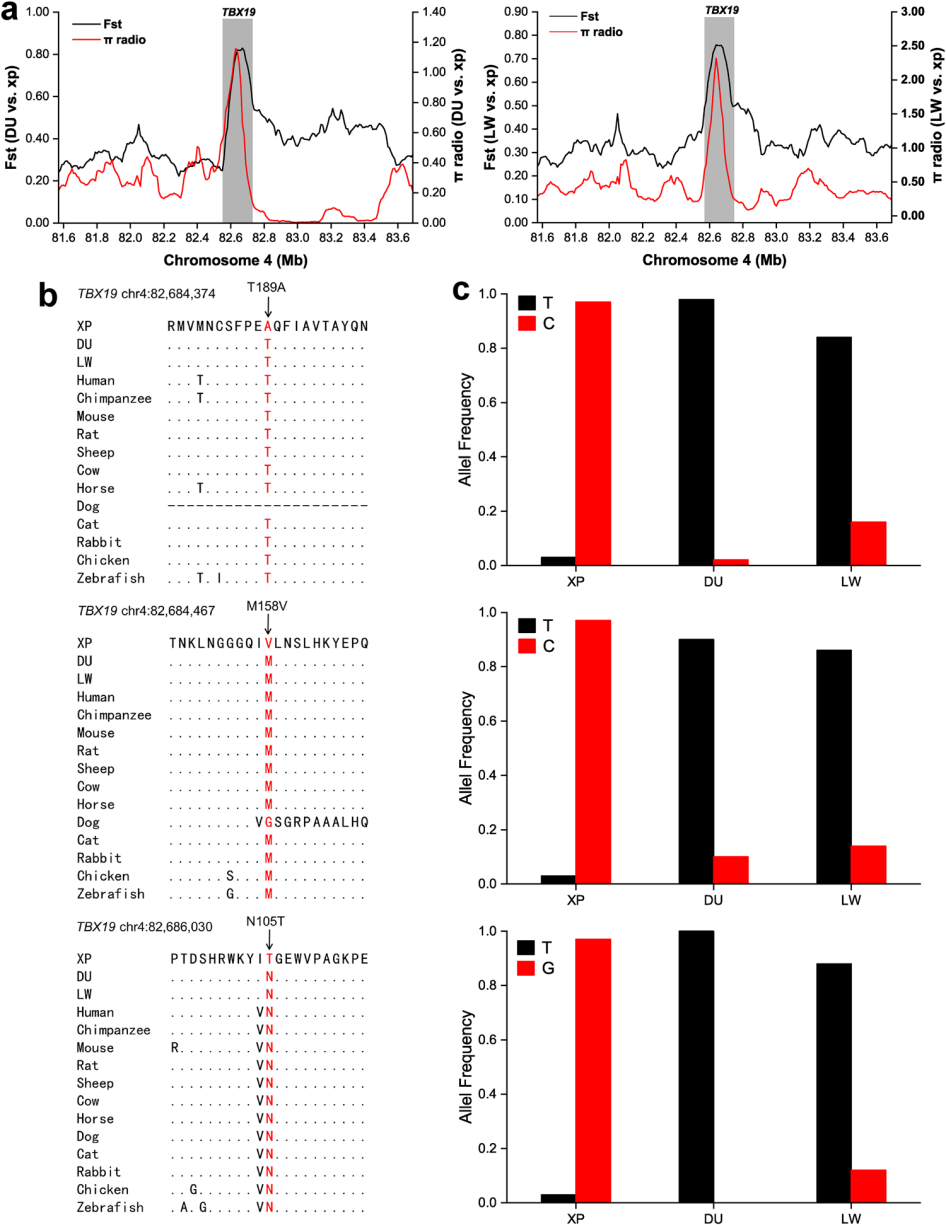
Characterization of selection signals around *TBX19* gene locus in XPs, and three putative functional variants (p.N105T, p.M158V, p.T189A). (**a**) The Fst and θπ values around the *TBX19* locus. (**b**) Cross-species alignment of the protein sequences around three functional variants (p.N105T, p.M158V, p.T189A) in *TBX19* gene. The dots in the alignment indicated the amino acids which were identical with those in XPs, and the dashes indicated the missing data. (**c**) Distribution of the allele frequency of the three variants in three populations (XP, DU, and LW).

In addition, 274 and 120 genes were shared between two and three comparisons, respectively (Fig. 5b, Table S4). These genes were associated with multiple phenotype terms (Table 2), such as growth and development processes (*IGF1R*, *PROP1*, *TBX19*, *STAC3*, *RLF*, *SELENOM*, *MSTN*), immune response (*SERPINB2*, *ADGRE5*, *STAT6*, *IL2*, *CD80*, *RHBDD3*, *PIK3IP1*), environmental adaptation (*NR2E1*, *SERPINB8*, *SERPINB10*, *UVSSA*, *EXPH5*, *VEGFC*), reproduction (*CCNB2*, *TRPM6*, *EYA3*, *CYP7B1*, *LIMK2*, *RSPO1*, *ADAM32*, *SPAG16*), and meat quality traits (*DECR1*, *EWSR1*)^6,12–41^*.* In particularly, *TBX19* on SSC4 showed high Fst and θπ values compared with neighboring regions in the DU vs XP and LW vs XP comparisons (Fig. 9a), and *LIMK2* on SSC14 was also strongly selected in these two comparisons (Fig. S3b). *EYA3* on SSC6 was strongly selected in the LW vs XP and MS vs XP comparisons (Fig. S3a). *ADAM32* on SSC15 showed significant signals in the TT vs XP and MS vs XP comparisons (Fig. S3c). For *TBX19*, 364 highly differentiated SNPs in the upstream, downstream, UTR5, UTR3, exon, and intron regions were simultaneously distributed in both DU vs XP and LW vs XP comparisons (Table S7). Among 364 SNPs, 13 SNPs were observed in five exons and six of these were nonsynonymous variants (Table S8). Furthermore, we found that three nonsynonymous variants (p.N105T, p.M158V, p.T189A) were predicted as functional-altering variants (SIFT < 0.05) (Fig. 9c, Table S8), and they were highly conserved among multiple vertebrate species (Fig. 9b). Further analysis of *LIMK2* gene showed that 115 homozygous SNPs had already been fixed in XPs (Table S9).

**Table 2:** A partial list of candidate genes with previous evidences that were shared among two or three comparisons datasets.

Chr	Start	End	Candidate gene	Comparisons	Gene function	References
1	74,271,042	74,292,681	NR2E1	TT vs XP/LW vs XP	Behavioral defense response	^12^
1	112,907,047	112,942,219	CCNB2	TT vs XP/DU vs XP/LW vs XP	Litter size trait	^13^
1	137,387,925	137,690,666	IGF1R	TT vs XP/LW vs XP	Growth Traits	^14^
1	157,820,645	157,836,904	SERPINB8	TT vs XP/LW vs XP	Environmental adaptation	^15^
1	157,841,510	157,866,913	SERPINB10	TT vs XP/LW vs XP	Implicated in UV-induced stress	^16^
1	157,872,212	157,886,753	SERPINB2	TT vs XP/LW vs XP	Immune response	^17^
1	227,811,204	227,963,110	TRPM6	DU vs XP/MS vs XP	Litter size trait	^18^
2	64,844,792	64,890,939	ADGRE5	LW vs XP/MS vs XP	An activation marker on T cells	^19^
2	79,627,603	79,631,270	PROP1	TT vs XP/MS vs XP/LW vs XP	Growth traits	^20^
4	46,730,461	46,767,427	DECR1	TT vs XP//MS vs XP/LW vs XP	Carcass and meat quality, lipid composition	^21^
4	69,616,035	69,806,496	CYP7B1	MS vs XP/DU vs XP/LW vs XP	Male reproductive behavior	^22^
4	82,663,581	82,697,813	TBX19	DU vs XP/LW vs XP	Development and growth	^23^
5	22,406,364	22,421,990	STAT6	MS vs XP/DU vs XP/LW vs XP	Immune response	^24^
5	22,549,008	22,556,194	STAC3	DU vs XP/LW vs XP	Vertebrate skeletal muscle development	^25^
6	85,095,409	85,185,799	EYA3	LW vs XP/MS vs XP	Male and female seasonal estrus	^26,27^
6	93,665,138	93,685,197	RSPO1	TT vs XP/MS vs XP	Sex development	^28^
6	95,900,086	95,994,044	RLF	TT vs XP/MS vs XP/LW vs XP	Muscle development and growth	^29^
8	573,421	601,944	UVSSA	TT vs XP/DU vs XP/LW vs XP	Environmental adaptation	^30^
8	101,640,944	101,645,609	IL2	TT vs XP/MS vs XP/DU vs XP	T-cell growth factor	^31^
9	36,885,212	37,006,396	EXPH5	TT vs XP/DU vs XP/LW vs XP	Adaptation to Arctic or Antarctic environments	^32^
13	140,693,382	140,728,980	CD80	DU vs XP/LW vs XP	T cell activation	^33^
14	46,400,026	46,408,366	RHBDD3	DU vs XP/LW vs XP	Suppress the production of IL-6	^34^
14	46,408,053	46,442,086	EWSR1	DU vs XP/LW vs XP	Intramuscular fat	^35^
14	47,902,860	47,905,440	SELENOM	DU vs XP/LW vs XP	Growth trait	^36^
14	47,946,396	48,040,822	LIMK2	DU vs XP/LW vs XP	Male infertility	^37^
14	48,040,881	48,051,660	PIK3IP1	DU vs XP/LW vs XP	Inhibition of T-cell activation	^38^
15	39,037,893	39,133,041	VEGFC	TT vs XP/MS vs XP	High-altitude adaptation	^6^
15	47,388,300	47,520,236	ADAM32	TT vs XP/MS vs XP	Male reproduction	^39^
15	94,620,269	94,628,546	MSTN	DU vs XP/LW vs XP	Inhibit of skeletal muscle development and growth	^40^
15	115,809,180	116,760,790	SPAG16	TT vs XP/DU vs XP	Male reproductive function	^41^

The absolute allele frequency difference (ΔAF) for each SNP was calculated in each comparison. As result, we identified 5885, 62,332, 855,864 and 483,774 SNPs with the criteria of AF_Xiang_ > 80% and AF _non-Xiang_ < 20%, which located within 1466, 4937, 13,518, and 10,220 genes from comparisons between XP and TT, MS, DU, and LW, respectively (Table S10).

Next, we evaluated the overlap between the candidate genes in Fst-θπ analysis and those detected genes in the absolute allele frequency difference (ΔAF) analysis in each comparison. In total, we obtained 174, 132, 186, and 308 genes overlapped among the results from Fst-θπ and ΔAF analysis in the TT vs XP, MS vs XP, DU vs XP, and LW vs XP comparisons, respectively (Table S11). Many of these overlapped genes have already been mentioned above, such as *ZCCHC2* and *TBX19*. Especially the *ZCCHC2* gene, it was commonly identified by Fst-θπ and ΔAF analysis in all four comparisons. These results supported by Fst, θπ and ΔAF methods further indicated the reliability of the highlighted genes in this study.

Considering the impact of nonsynonymous SNPs (nsSNPs) on protein function, we further investigated the genes detected only by ΔAF analysis that contain missense variants. In total, we explored 143 missense variants with marked allele frequency differences distributed in 89 functional genes that might affect domestication or phenotypic traits of XPs among all four comparisons (Table S12). For example, we identified two missense mutations with the highest ΔAF (ΔAF = 1) occurring in *NR6A1* and *LTBP2* in both DU vs XP and LW vs XP comparisons. The first was a c.T485C (p.L162P, rs326780270) substitution in *NR6A1* (nuclear receptor subfamily 6 group A member 1), which encodes an orphan nuclear receptor and has been considered a strong candidate for affecting the numbers of vertebrae in swine^42^. The other was a c.T2732C (p.M911T, rs328662847) substitution in *LTBP2* (latent transforming growth factor beta binding protein 2) that are associated with thoracic vertebrae numbers in the pig population^43^. Our results indicated that both of the two genes might play important roles in the vertebrae development of Xiang pigs. Moreover, we detected other important missense variants with marked allele frequency differences occurring in the functional genes. All of the strongly differentiated missense variants identified from the four comparisons were presented in Table S12.

### GO terms and KEGG pathway enrichment analyses

**Figure 10:**
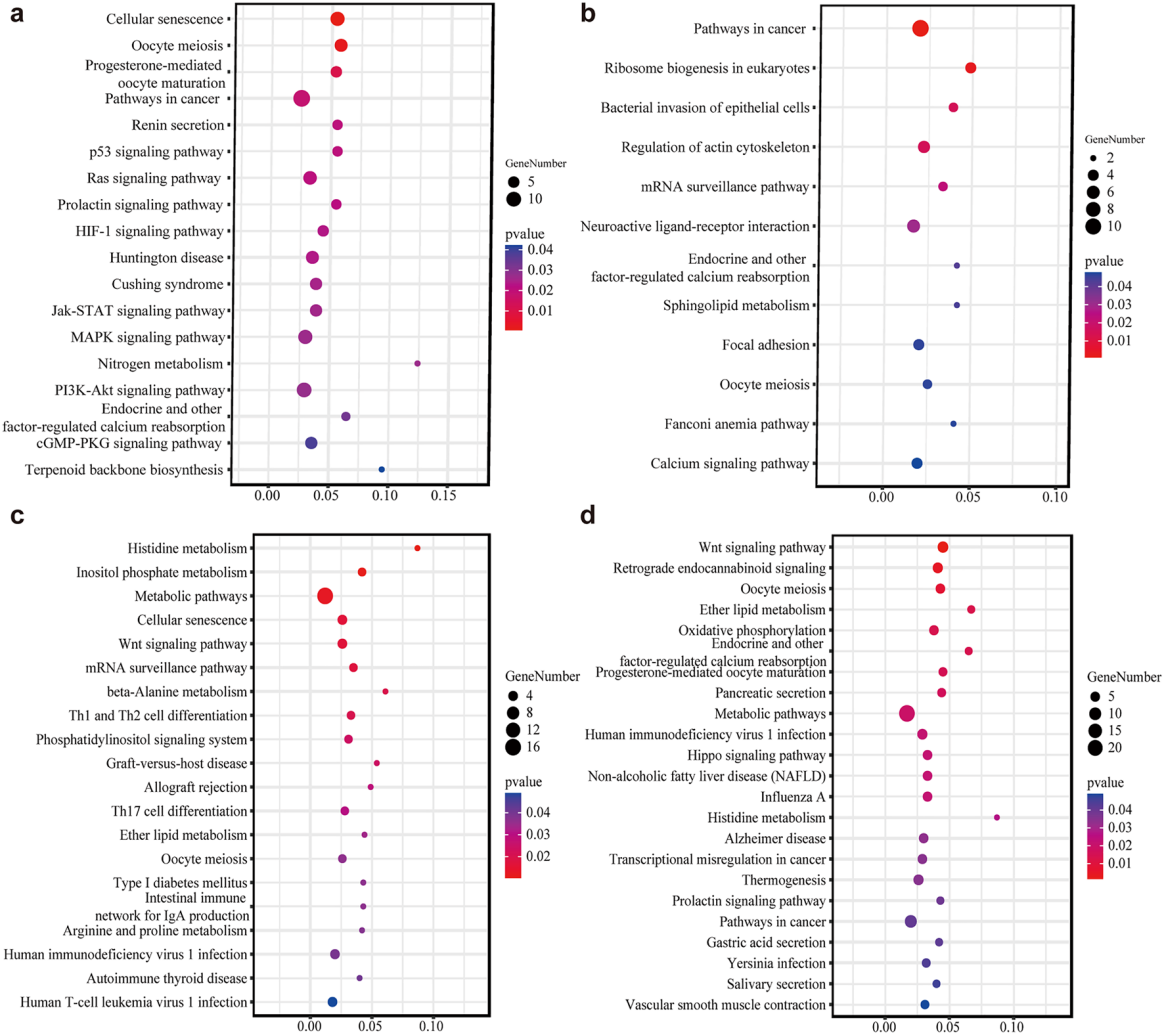
The significant KEGG pathway enrichment of the candidate genes under selection in XPs. (**a**) TT vs XP, (**b**) MS vs XP (**c**) DU vs XP, and (**d**) LW vs XP. Note: KEGG pathways were analyzed via KEGG database (https://www.kegg.jp/kegg/kegg1.html).

Subsequently, we searched for significantly overrepresented (*P*-value < 0.05) GO terms and KEGG pathways related to the candidate genes with gene symbols identified from both Fst and θπ statistics in each comparison. In the TT vs XP comparison, 427 candidate genes under selection were used for the GO and KEGG analysis, which resulted in 224 significant GO terms and 18 statistically significant KEGG terms. The top three most significant KEGG terms included cellular senescence, oocyte meiosis, and progesterone-mediated oocyte maturation terms (Fig. 10a). Among the 224 significant GO categories, we detected the terms involved in adaption, including “positive regulation of bone resorption”, “locomotory behavior”, “actin cytoskeleton”, “skin development”, “sensory perception of sound”, “actin cytoskeleton”, “actin cytoskeleton reorganization” (Table S13-1). In the MS vs XP comparison, 199 selected genes resulted in 187 significantly enriched GO terms (Table S13-2) and 12 KEGG pathways (Fig. 10b). Among the significantly enriched GO categories, the highest counts were associated with the immune system. The 12 KEGG enrichment pathways were significantly enriched in pathways involving cancer, bacterial invasion of epithelial cells, Fanconi anemia pathway, calcium signaling pathway, and other important biological processes. For the comparison between XPs and DUs, 180 selected genes resulted in 161 significant GO terms (Table S13-3) and 20 KEGG enrichment terms (Fig. 10c). The top three most significant terms were associated with the nucleus (GO:0005634; *p*-value = 1.72E−08), nucleoplasm (GO:0005654; *p*-value = 3.65E−06), and cytoplasm (GO:0005737; *p*-value = 1.08E−05) terms. In addition, some of these significantly enriched terms were related to morphogenesis and development. Disease-resistance related processes displayed the greatest KEGG enrichments, such as Th1 and Th2 cell differentiation, graft-versus-host disease, Th17 cell differentiation, and intestinal immune network for IgA production. Six enriched terms were related to metabolism. In the comparison group of XPs and LWs, 307 selected genes were significantly enriched in 128 GO terms (Table S13-4) and 23 KEGG path (Fig. 10d). Of which, eight GO categories were associated with muscle development and growth, including sarcomere, actin filament binding, actin cytoskeleton, actin monomer binding, actin binding, filamentous actin, negative regulation of cell growth, and regulation of growth. Moreover, two GO categories were involved in male reproduction, including spermatogenesis, sperm axoneme assembly and fertilization. The Wnt signaling pathway displayed the greatest KEGG enrichment. The remaining enriched pathways were primarily related to metabolism, secretion and disease (Fig. 10d).

## Discussion

It is well known that pig is one of the first domesticated animals. During the process of domestication and breeding, natural and artificial selection caused many changes of pig in body conformation, production performance, immune function, and adaptability to the environment^44^. The distinct imprints have been left in the genomes of domestic pigs. In recent years, selection signatures have been used to elucidate the genetic basis of many complex traits in agricultural animals^5,6^. In the present study, we performed the whole-genome resequencing of 25 unrelated individual Xiang pigs, and conducted population structure and LD analysis for the Xiang pig and other four breeds (i.e., Tibetan, Meishan, Duroc and Yorkshire). Our analyses revealed the population structure was mostly correlated with the geographic distribution. Xiang pigs displayed lower LD decay, indicating that Xiang pigs are less affected by artificial selection than the European and Meishan pig breeds. In addition, we explored the selection signatures that help provide potential genomic evidence linking the domestication of Xiang pigs with their breed characteristics.

To screen genome-wide regions under positive selection in Xiang pigs, we first selected windows with the top 5% of Fst and θπ values and identified 38 genes in the intersection of all four comparisons. Of those, seven genes (*ZCCHC2*, *SLC26A7*, *SDR9C7*, *RDH16*, *TAC3*, *MYO1A*, *LYZ*, and *PDE1A*) were involved in important biological processes. Except for *PDE1A*, other seven selection signatures were also confirmed by ΔAF analysis. *ZCCHC2,* as a member of the zinc finger CCHC-type (ZCCHC) superfamily protein, has nucleic acid–binding properties. Several literature have implicated the *ZCCHC2* is correlated with diverse diseases. For example, ZCCHC2 participates in the regulation of retinoblastoma tumorigenesis by suppressing the activity of c-Myc^45^. A SNP in *ZCCHC2* is associated with insect bite hypersensitivity in Exmoor ponies^46^ Besides, *ZCCHC2* has previously been identified as important gene contributing to the benign (BEN) phenotypes of SIV/HIV infections, suggesting its role in the host defense against virus infections^47^. Intriguingly, *ZCCHC2* showed high Fst and θπ scores in all four comparisons, implicating that *ZCCHC2* gene could have been preferentially selected in XP. Our findings supported that *ZCCHC2* likely played roles in disease resistance process of XP. *SLC26A7* on SSC4 is one of the sulfate/anion transporter gene families and involves in the regulation of intracellular pH through chloride channels. It has been identified as a promising candidate gene for plateau adaptability in Tibetan pigs^48^. Interestingly, we found that four neighbor protein-coding genes (*SDR9C7*, *RDH16*, *TAC3* and *MYO1A*) on SSC5 (22.22–22.36 Mb) were also under selection in XP. *SDR9C7* is a well‐recognized gene for skin barrier formation due to its role in dehydrogenation of acylceramides^49^. *RDH16*, participating in energy and metabolism processes in adipose tissues in pigs and rats, has been shown to be one of the functional genes involved in the body size of Guizhou small goats^50^. Thus, it was reasonable to predict that *RDH16* may be a promising candidate gene associated with the body size of the XP. *TAC3* encoding neurokinin B (NKB) plays an important role in regulating the human reproductive axis. A previous study has demonstrated that TAC3 is a key regulator of kisspeptin expression and GnRH pulse secretion^51^. A distinctive finding indicate that the variation of A63P in *TAC3* may increase the risk of earlier puberty onset in Chinese girls^52^. The results indicated that *TAC3* variants in XP, especially one nonsynonymous variants and one functional-altering variant, may contribute to sexual maturity of XP by regulating kisspeptin expression and GnRH secretion. *MYO1A* (myosin IA) is a candidate gene that lightens the coat color of cattle, which contributes to heat stress resistance in cattle^53^. In humans, variants in the *MYO1A* gene are reported in non-syndromic sensorineural hearing loss^54^. In the present study, we detected a novel missense variant/splice variant (p.V299I) in *MYO1A.* The splice variant may affect *MYO1A* expression. This led us to hypothesize that the p.V299IA splice variant may have a role in local adaptation by regulating coat color and/or hearing in Xiang pigs. *LYZ* is an antibacterial enzyme which damages bacterial cell walls by catalyzing the hydrolysis of the b-(1,4)-glycosidic linkage between N-acetylglucosamine and the muramic acid of the peptidoglycan layer. LYZ displays antimicrobial, antitumoral and immunomodulatory properties, which have been extensively studied^55,56^. *PDE1A* are reported to be one of the hypoxia-related genes in Tibetan pig^6^.

To accurately detect the positively selected genes under selection in Xiang pig, we further explored the overlapped selected genes between two or three comparisons datasets. We identified seven genes implicated in growth and development processes (*IGF1R*, *PROP1*, *TBX19*, *STAC3*, *RLF*, *SELENOM*, *MSTN*). In *TBX19*, we simultaneously observed strong selective sweep signals (Fig. 9a) and 364 high-ΔAF SNPs in the DU vs XP and LW vs XP comparisons (Table S7). Among the list of these high-ΔAF SNPs, three missense mutations (N105T, M158V, and T189A) were within the evolutionary conserved T-box domain and were predicted as function-altering variants (Fig. 9b). This result was well consistent with that found in Chinese domestic pigs^23^. TBX19 (TPIT) functions by activating pituitary cell differentiation, and is also a positive regulator for pro-opiomelanocortin (*POMC*) expression, which contributes to the production of the adrenocorticotropic hormone (ACTH) in the hypothalamic–pituitary–adrenal (HPA) axis^57^. Mutations in this gene may lead to the isolated deficiency of pituitary POMC-derived ACTH^57,58^. Patients with ACTH deficiency are clinically characterized by severe obesity, weight loss, lack of appetite, hypoglycaemia and low blood pressure^58^. Previous studies have reported that this gene is related to development and growth in Chinese native pigs, such as Enshi black pig, Bama Xiang^59,60^. Together with these findings, *TBX19* may act as a candidate gene associated with slow development and growth trait in XP. Studies have revealed that *IGF1R* is associated with growth animal growth and development, such as Hulun Buir Sheep^14^ and European pigs^4^. PROP1 is a homeodomain transcription factor that controls the development of the anterior pituitary gland. Mutations within this gene caused dwarfism in mice and humans due to deficiency of pituitary hormone^61^. Numerous reports have demonstrated association of *PROP1* with growth traits in livestock, such as cattle^20^, sheep^62^, and horse^63^. *STAC3* is specifically expressed in tissues of skeletal muscle as a nutrient regulated gene and plays a role in vertebrate skeletal muscle development and function^25^. *RLF* has been reported to markedly enhance DNA methylation of factors related to transcriptional regulation, which plays an essential role in embryonic muscle development^29^. *SELENOM* has been identified as a candidate gene for growth traits in the Anqing six-end-white pig (ASP)^36^. MSTN (GDF-8) plays a negative role in regulating the formation and differentiation of muscle cells and subsequently inhibits the growth of skeletal muscles^40^. Mutations or deletions in *MSTN* can cause the proliferation and hypertrophy of muscle cells and muscle fibers. Interestingly, Bama mini-pigs have a higher level of *MSTN* mRNA than Landrace pigs^64^. Similarly, XP is also a mini-type breed. However, further studies are needed to confirm the expression level of *MSTN* in XP. Therefore, these genes detailed above may be the most promising genomic evidence that explain the small body size of Xiang pigs. Xiang pigs inhabit a remote mountainous area in Guizhou Province, where is known for its high elevation and high humidity. During long-term adaptation to the harsh environments, Xiang pigs have evolved their unique adaptive characteristics. By combining the results from Fst and θπ statistical analyses, we identified seven genes that participate in the immune response (*SERPINB2*, *ADGRE5, STAT6*, *IL2*, *CD80*, *RHBDD3*, *PIK3IP1*)^17,19,24,31,33,34,38^, which may be involved in shaping the particular disease-resistance characteristics of Xiang pigs. Additionally, we also uncovered genes, which may play different roles in adaptation processes of Xiang pigs. These genes include *NR2E1* for behavioral defense response^12^, *SERPINB8* for maintaining the mechanical stability of skin^15^, *SERPINB10* involved in the UV-induced cellular response^16^, *UVSSA* for response to ultra-violet (UV) radiation^30^, *VEGFC* responsible for regulating oxygen homeostasis^6^. The positive selection signatures identified here provided new insights into the potential adaptive mechanisms in XP. Apart from these findings associated with growth, immunity, and environmental adaptation, some important functional genes associated with reproduction (*CCNB2, TRPM6, EYA3, CYP7B1, LIMK2, RSPO1, ADAM32, SPAG16*)^13,18,22,26,26–28,39,41^ and meat quality (*DECR1, EWSR1*)^21,35^ were also identified under selection pressure in Xiang pigs (Table 2). Of these, *EYA3, LIMK2* and *ADAM32* showed particularly strong signs of selective sweeps presumably associated with the domestication of Xiang pig. *EYA3* (eyes absent 3) is an ancient retinal-determining gene, and its expression is induced by long-day stimulation in mammals. It is clear that EYA3 is an essential factor for modulating the expression of the TSHβ, which regulates changes in seasonal reproductive biology^26^. It has been reported that *EYA3* is an important candidate gene in male and female seasonal estrus^27^. *LIMK2*, especially the testis-specific isoform tLIMK2, is essential for the proper progression of spermatogenesis. Additionally, *LIMK1/2* has shown to participate in mouse embryo early cleavage and blastocyst formation by regulating the phosphorylation level of cofilin in mouse embryos^65^. In humans, three deleterious nonsynonymous substitutions in *LIMK2* are involved in male infertility^37^. When comparing with the European commercial breeds (DU and LW), *LIMK2* showed strong signs of selective sweeps in XP. Notably, the derived alleles of 115 SNPs in *LIMK2* exhibited high AF (AF = 1) in XPs, whereas these derived alleles displayed low AF (AF < 0.35) in European breeds (DU and LW) (Table S10). *ADAM32* has been identified as contributing to fertilization in mouse^39^. Compared with the MS, DU and LW breeds, XP exhibited lower prolificacy. Therefore, *EYA3, LIMK2* and *ADAM32* genes might be promising candidate genes involved in the reproduction traits of Xiang pig.

Based on the genome-wide SNP allele frequency (AF) analysis, we identified a suite of promising genes. We further found that some of these genes from ΔAF test colocalized with the selected regions detected in the Fst-θπ analysis (Table S11). These overlapped candidate genes may have undergone strong selection, and potentially affected phenotypic traits of Xiang pig, such as *ZZHC2*, *LIMK2* and *TBX19* mentioned above. Apart from the overlapped genes, we further focused specifically on the candidate genes detected only in the ΔAF analysis that contained missense variants. Among these candidate genes, two well-known genes (*NR6A1*, *LTBP2*) were of particular significance, as they are both involved in affecting the numbers of vertebrae in swine. The *NR6A1* (nuclear receptor subfamily 6, group A, member 1) gene is also known as the germ cell nucleus factor (GCNF). Thus far, a large number of studies have confirmed the association between *NR6A1* gene polymorphisms and lumbar vertebrae numbers in pigs and other species^42,66^. In the present study, one nonsynonymous mutation (c.T485C, rs326780270) was identified in the coding region of exon-4 of *NR6A1* gene. The derived C allele frequency at this SNP locus was 1.0 in XP, whereas it was 0.0 in the European breeds (DU and LW). *LTBP2* is expressed prominently in the outer lamellar layers of the annulus fibrosus of the fetal intervertebral disk in humans^67^. LTBP2 regulates the activity of growth differentiation factor Gdf11 by inhibiting the extracellular processing of proGdf11. Gdf11 knock-out mice exhibit an increase in the numbers of ribs from 13 (wild type) to 18^68^. SNP (c.4481A > C) in LTBP2 is associated with thoracic vertebrae numbers in an F2 intercross population between Landrace and Korean native pigs^43^. In our study, we observed one nonsynonymous mutation (c.T2732C, rs328662847) in the coding region of exon-17 of *LTBP2* gene. The derived C allele frequency at this SNP locus was 1.0 in XP, whereas it was 0.0 in the European breeds (DU and LW). Given the importance of *NR6A1* and *LTBP2* to vertebrae numbers, the two SNPs: rs326780270: T > C in NR6A1 and rs328662847: T > C, may affect vertebrae development in XP.

For each comparison, we subsequently investigated the functions associated with the annotated genes from both Fst and θπ statistical approaches by analyzing over-represented GO term and KEGG pathway analysis. In the TT vs XP comparison, the most significant pathway was “Cellular senescence” (Fig. 10a), which is often regarded as an anti-cancer mechanism^69^. In the MS vs XP comparison, the KEGG analysis revealed that “pathways in cancer” represented the greatest enrichment. We also detected calcium signaling pathway, which plays a crucial role in muscle function and plasticity and is involved in many processes during animal embryonic development. Of note, 15 significant GO terms related to the immune system in XP were identified, such as the adaptive immune response, response to virus, toll-like receptor 2 signaling pathway, and interleukin-2 receptor binding (Table S13-2). Other GO terms of interest were related to adaptability, including aggresomal, locomotor behavior, cellular response to heat, cellular response to UV. The results were consistent with the fact that XP exhibits strong disease resistance and high adaptability. In the DU vs XP comparison, the results of KEGG enrichment analysis (Fig. 10c) revealed that eight pathways involved in the immune system or infectious diseases were significantly enriched, including Th1 and Th2 cell differentiation, graft-versus-host disease, allograft rejection, Th17 cell differentiation, intestinal immune network for IgA production, human immunodeficiency virus 1 infection, autoimmune thyroid disease, and human T-cell leukemia virus 1 infection. This may provide a better mechanism for the disease resistance in XP. Compared with Duroc pigs, Xiang pigs are smaller and not as tall. We identified seven significant pathways that were related to developmental and metabolic processes including Wnt signaling pathway, histidine metabolism, inositol phosphate metabolism, metabolic pathways, beta-alanine metabolism, ether lipid metabolism, and arginine and proline metabolism. Among the significant enriched GO categories (Table S13-3), eight terms (GO:0060612 GO:0048536 GO:0001889 GO:0003143 GO:0061303 GO:0055013 GO:0060430 GO:0060322) were associated with development processes. These results indicated that differences in development and metabolism between XPs and DUs. In the LW vs XP comparison, the most significant pathway was “Wnt signaling pathway” (Fig. 10d). Wnt signaling pathway participates in many developmental events during embryogenesis and is essential for muscle fiber growth and maintenance^58,70^. Wnt signaling pathway is also involved in satellite cell proliferation and differentiation during adult skeletal muscle regeneration^71^. We also found significantly over-represented GO terms associated with male reproduction (GO:0007283 GO:0007288 GO:0009566) and growth (GO:0030308 GO:0040008) (Table S13-4). Our findings reflect that the candidate genes are mainly involved in disease resistance, adaptability, and developmental and metabolic processes in XPs. The largest number of terms related to disease resistance and adaptation, which further indicate that Xiang pigs underwent natural selection pressure to adapt to their environments.

## Conclusions

We conducted full resequencing of 25 Xiang pigs individually, resulting in a comprehensive whole-genome map and the identification of 21 M heritable SNPs. Moreover, we detected the genomic signatures and found that the genes positively selected in Xiang pigs were involved in crucial biological processes. We highlighted some genes/regions under possible selection relevant to disease resistance (*ZCCHC2*), early maturation (*TAC3*), reproduction (*LIMK2, EYA3, ADAM32*) and growth (*TBX19*). We mainly exemplified striking evidence of selection at *ZCCHC2* and *TBX19*, which are correlated with diseases, and growth and early development, respectively. Additionally, important missense mutations with high ΔAF were identified within *NR6A1* and *LTBP2* genes that mainly contribute to vertebrae numbers of Xiang pigs. These findings will facilitate the understanding of the germplasm characteristics and support further investigation of the mechanisms underlying selection in XP pig breed.

## Materials and methods

### Ethics statement

All animal procedures were approved by Guizhou University Subcommittee of Experimental Animal Ethics (EAE-GIU-2021-P009) and were conducted the rules of animal experimental ethics. The study is also in accordance with ARRIVE guidelines.

### Sample collection and sequencing

In this study, we obtained a total of 25 unrelated Xiang pigs (XPs) based on pedigree records from the pig farm in Congjiang County, Guizhou Province. For each pig, the whole blood sample was collected for DNA extraction. The high-quality DNA was used for the whole-genome resequencing. The Illumina DNA libraries (Paired-end, 2 × 150 bp) were constructed and sequenced on the Illumina Hiseq 2500 sequencing platform by BGI-Shenzhen, China. Additionally, we downloaded 100 publicly-available pig genome sequences from NCBI database, including Tibetan (TT, n = 25), Meishan (MS, n = 25), Duroc (DU, n = 25), and Yorkshire (LW, n = 25).

### Mapping, SNP calling and annotation

To avoid low-quality reads, the raw paired-end reads were filtered and trimmed using the NGSQC Toolkit with default parameters^16^. High-quality reads were then aligned to the Sscrofa11.1 reference sequence using the Burrows-Wheeler Aligner (BWA) with the “bwa-mem” algorithms^72^. SAMtools was used for sorting and indexing the aligned BAM files^73^. Potential duplicates were removed using Picard tools. Finally, we jointly used “HaplotypeCaller”, “CombineGVCFs” and “GenotypeGVCFs” with default parameters in GATK to call variants, which generated genotype calls in Variant Call Formats (VCF)^74^.

To exclude possible false positives, we filtered the variants according to the strict filter criteria. We removed those INDELs in the VCF file using the following options: QUAL < 30.0, QD < 5.0, FS > 200, ReadPosRankSum < − 20.0^75^. High-quality SNPs were identified according to the filtering criteria: QUAL > 30, OD > 5.0, FS < 60.0, MQ > 40.0, MQRankSum > − 12.5, ReadPosRankSum > − 8.0^75^. Variants that passed quality control filter were functionally annotated with the ANNOVAR softwares^76^ based on the corresponding pig genome annotation file from the Ensembl database.

### Population structure and linkage disequilibrium

To better understand the genetic relationships among the four pig populations in our study, we performed PCA, NJ tree and ADMIXTURE analysis. Principal component analysis (PCA) was carried out using the GCTA software^77^, and the first two eigenvectors were plotted in the ggplot2 package under the R platform. For phylogenetic tree analysis, PLINK was used to calculate genetic distance matrix^78^. Next, the phylogenetic tree was constructed with the neighbor joining (NJ) algorithm in MEGA^79^ and displayed with FigTree^80^. Moreover, the population ancestry was estimated using ADMIXTURE software^81^with K set from 2 to 5. The genome-wide linkage disequilibrium (LD) decay between pairwise SNPs was assessed and visualized using PopLDdecay software^82^.

### Selective sweeps and functional enrichment analyses

Before detecting selection signals, we filtered the SNPs with minor allele frequency (MAF) < 0.05 and call rates < 0.90 and excluded sites with a missing rate > 20% using PLINK. In order to identify the candidate regions under positive selection in XPs, we first calculated the fixation statistics (Fst ) and population nucleotide diversity ratio (θπ) according to the the procedure described by Li et al.^6^. Both the Fst and θπ statistics require the usage of the standard genotype data. The Fst value was estimated based on the differences in allelic frequencies between populations^83^. This method is effective for identifying the genomic pattern affected by the divergent selection^84^. Domestication and selection resulted in decreased nucleotide diversity in populations. Thus, the method based on θπ can serve as efficient statistic to identify signatures of selection in populations^6,74^. In fact, numerous studies have been performed to detect the selective signal sweep regions in animals by using both Fst and θπ methods^6,75,85^. The average Fst and θπ were calculated by VCFtools (v0.1.13) in 100-kb sliding windows with a 10-kb step size^6,85^ between XP and the other four control breeds. To avoid spurious selection signals, windows containing less than 10 SNPs were discarded. Putative selection targets were identified as the candidate regions in fully overlapping windows with high Fst (Fst > 95%) and θπ (θπ > 95%) values^85^. The selected regions were annotated using Bedtools (v2.17.0).

The highly differentiated SNPs are likely to be directly targeted by selection or to occur near loci under selection^86^. Therefore, we also estimated allele frequency of single SNP with a genome scan for each pig population and measured the absolute allele frequency difference (ΔAF) to further detect putative selection signatures^5^. The ΔAF method is best suited for detection of fixed or approximately fixed alleles in Xiang pigs^87^. The ΔAF value per SNP between the Xiang population and the other four pig populations was calculated using the formulas: ΔAF = abs (AltAF_XP _− AltAF_TT_), ΔAF = abs (AltAF_XP _− AltAF_MS_), ΔAF = abs (AltAF_XP _− AltAF_DU_), and ΔAF = abs (AltAF_XP _− AltAF_LW_), respectively. After comparisons of ΔAF value between Xiang and each of the other four groups, we searched for Xiang-specific SNPs across the entire genome with the criteria of AF_Xiang_ > 80% and AF _non-Xiang_ < 20%^88^. By appliying the absolute allele frequency difference (ΔAF) analysis, we could not only furthe evaluate the reliability of the results from Fst and θπ statistics, but also mine the potational selected gene containg interesting candidate mutations.

To investigate the biological enrichment of genes under selective pressure, KEGG pathway and GO classes were analyzed based on the candidated genes from Fst and θπ methods by using KOBAS (v3.0)^89^. The terms with *p*-value smaller than 0.05 were considered to be statistically significant.

## Supplementary Information

Supplementary Information 1.

Supplementary Table S1.

Supplementary Table S2.

Supplementary Table S3.

Supplementary Table S4.

Supplementary Table S5.

Supplementary Table S6.

Supplementary Table S7.

Supplementary Table S8.

Supplementary Table S9.

Supplementary Table S10.

Supplementary Table S11.

Supplementary Table S12.

Supplementary Table S13.
