# Supplementary material for: Whole-genome sequence analysis reveals selection signatures for important economic traits in Xiang pigs
Source: Sci Rep. 2022 Jul 12;12:11823. doi: 10.1038/s41598-022-14686-w (PMC9276726; doi:10.1038/s41598-022-14686-w)
Supplement: Supplementary file 1 — Supplementary Information 1. [file 41598_2022_14686_MOESM1_ESM.docx]

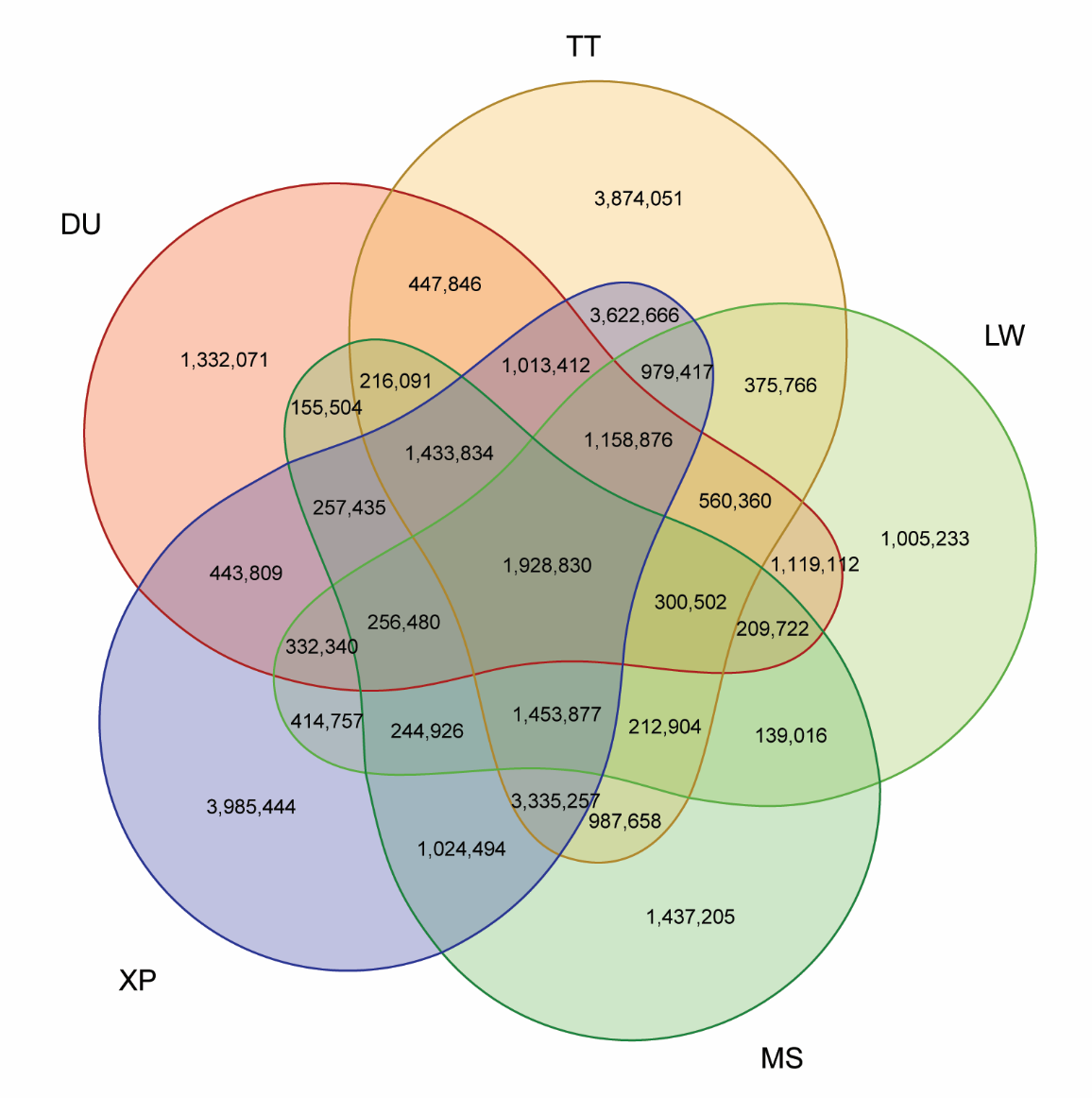


**Figure S1.** Venn diagram shows the numbers of unique and shared SNPs between XP, TT, MS, DU and LW populations.


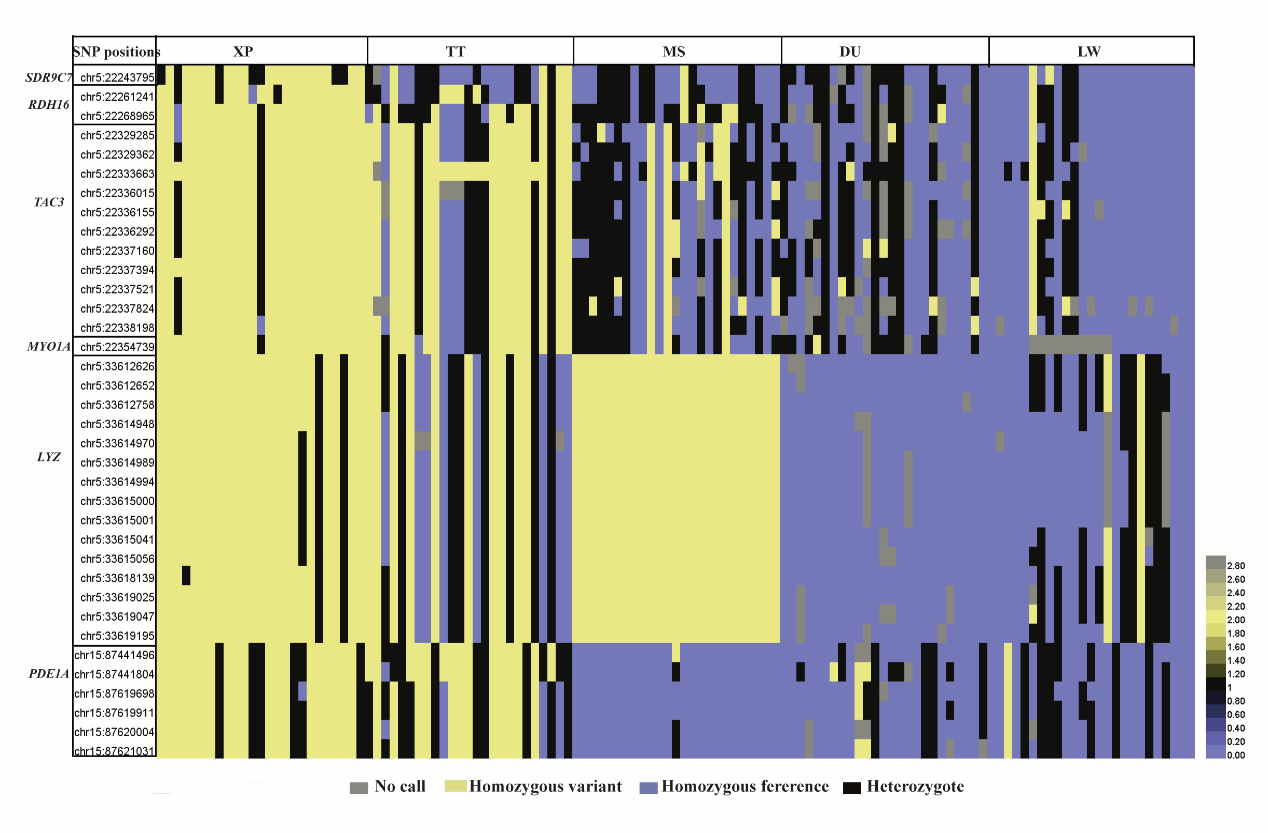


**Figure S2.** The genotyping results of 36 highly different SNP located in UTRs, exon, and downstream/upstream within *SDR9C7*, RDH16, *TAC3*, *MYO1A*, *LYZ* and *PDE1A* across five pig populations.


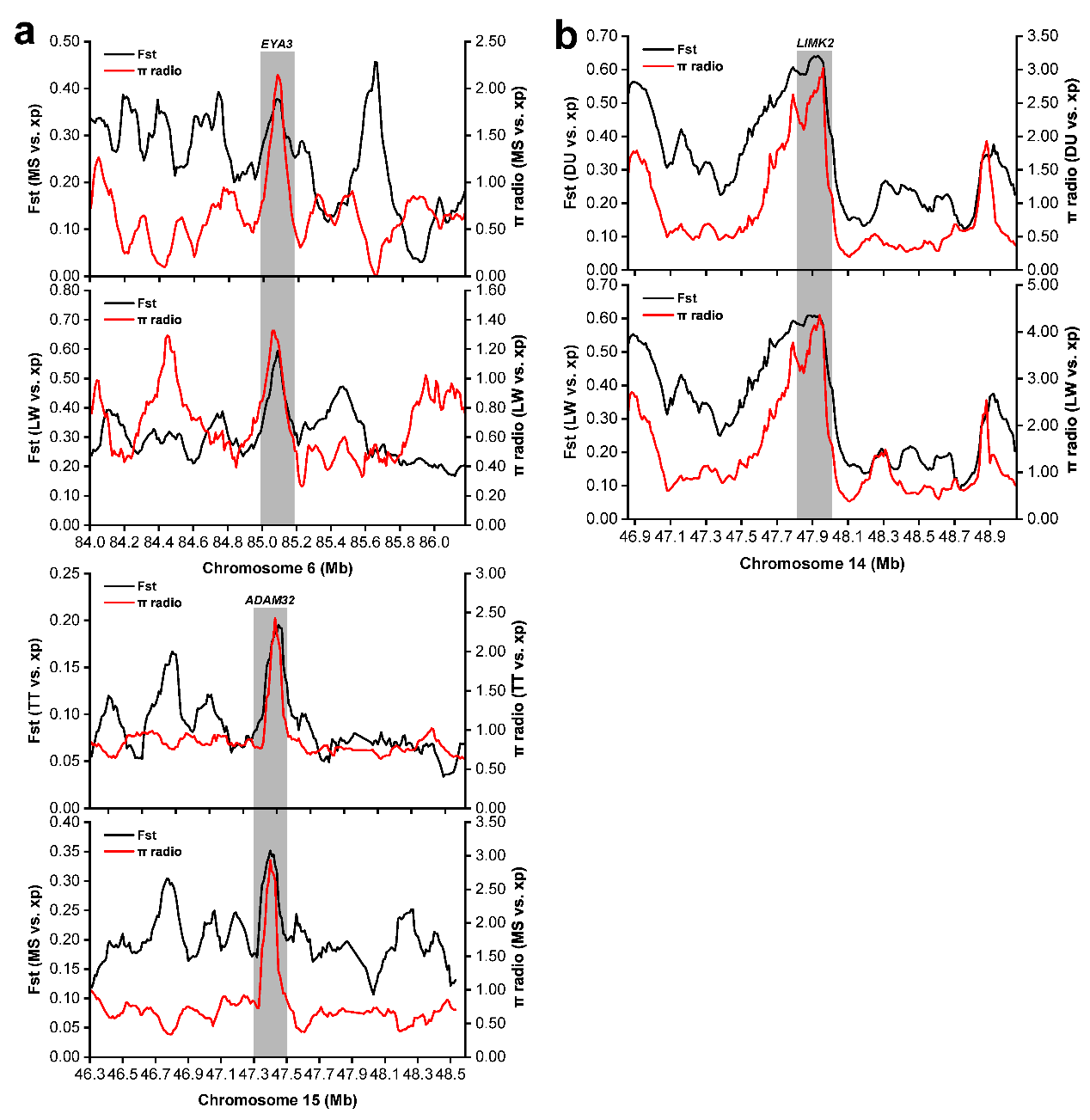


**Figure S3.** Characterization of selection signals around three genes locus in XP. (a) The Fst and θπ values around the *EYA3* locus. (b) The Fst and θπ values around the *LIMK2* locus. (c) The Fst and θπ values around the *ADAM32* locus.
